# O18 When joints vanish

**DOI:** 10.1093/rap/rkab067.017

**Published:** 2021-10-19

**Authors:** Lavanya Rajagopala, Chathurika Dandeniya, Shalene Janagan, Mahesh Gunathilaka, Hasanthi Perera, Dhanushka Karunarathna, Jayathri Jagoda

**Affiliations:** Lady Ridgeway Hospital for Children, Colombo, Sri Lanka

## Abstract

**Case report - Introduction:**

Paediatric rheumatology is an interesting speciality. Exact characterisation of juvenile idiopathic arthritis (JIA) and other autoinflammatory conditions still remains a grey area in rheumatology. To add to the confusion, a horde of heritable and acquired conditions can mimic these diseases, making diagnosis a real challenge.

Idiopathic multicentric carpotarsal osteolysis syndrome (MCTO) is a rare skeletal disorder characterised by aggressive osteolysis, predominantly carpal and tarsal bones. The early clinical presentation is joint pain, and can mimic polyarticular JIA. Progressive nephropathy in latter stages is a known association. Genetic tests helps in early diagnosis of MCTO.

**Case report - Case description:**

A 17-year-old male was managed as polyarticular JIA since the age of 1.5 years. He was the first child of healthy nonconsanguinous Sri Lankan parents. His prenatal and perinatal periods were uncomplicated. His development was unremarkable until joint symptoms evolved. The family history was insignificant for any bone disorder. He was on long-term methotrexate and approximately 15 monthly tocilizumab infusions but to no avail.

His distinctive facial features included cloudy cornea and subtle facial dysmorphism such as maxillary hypoplasia. Upper limb deformities included shortening of arms and forearms, fixed flexion deformity of the left elbow joint and distorted and grossly swollen wrists. Deformities of the lower extremities included short thighs and short bowed legs. A painful limp on the right was observed.

His inflammatory markers were persistently normal. His initial investigations revealed moderate proteinuria without renal impairment. Serum electrolytes, bone profile, ALP and PTH was unremarkable. This clinical case was revised due to the apparent resistance to treatment and uncharacteristic limb deformities.

A skeletal survey was performed. Radiographic examination revealed disappearing carpal and tarsal bones as well as disappearing long bone ends, compatible with a diagnosis of idiopathic multicentric osteolysis. The characteristic 'sucked candy' apperance of metacarpals was appreciated. In addition, severe cortical thinning of all bones indicating osteopenia was observed.

He was also noted to have proteinuria and was referred to the nephrologists for further evaluation.

The adolescent was taken off all immunosuppressive therapy without any clinical worsening. Given the limited evidence for treatment strategies, he was initiated on a trial of oral bisphosphonates. He responded well to treatment symptomatically, as pain gradually subsided. He was further referred for rehabilitation.

Genetic test for MAFB seqeuencing (V-maf musculoaponeurotic fibrosarcoma oncogene homolog B) was not performed due to unavailability.

This clinical case further directed us to diagnose multicentric carpo-tarsal osteolysis in two more children who were initially managed for JIA and had poorly responded to immunosuppressive therapies.

**Case report - Discussion:**

We report the challenges in diagnosing a mimicker of JIA, where awareness of the rare syndrome and high index of suspicion plays a pivotal role in the diagnosis of the condition, especially in the absence of genetic testing due to resource-poor settings.

MCTO, is a rare osteolytic condition largely of unknown etiology. The syndrome manifests with progressive loss of carpal and tarsal bones in childhood. Affected children have arthritic-like episodes, followed by progressive deformities, radiographic osteolytic changes, and variable degrees of disability. There are many different types of osteolytic syndromes with overlapping features and it may have a familial inheritance.

Clinical symptoms and signs occur early in childhood. They present with multiple bone and joint pains, and bony 'dysplasia' associated with reduced range of joint movements and functions. Our patient initially presented at the age of 2 years with features mimicking polyarticular JIA and these deformities developed gradually and were not noticed until adolescent stage.

Fragmentation, lysis and eventual disappearance of the distal ends of long bones, carpal and tarsal bones, is the hallmark feature. Studies demonstrate the presence of localised osteoclastic activity adjacent to osteolytic areas.

Radiological investigations help aid in diagnosis. The findings include characteristic osteolytic changes. Serological and histological investigations are always negative for classical inflammatory or autoimmune causes.

Described associations include protein losing nephropathy due to underlying focal segmental glomerulosclerosis, cloudy cornea and subtle facial dysmorphisms such as triangular face, maxillary hypoplasia and micrognathia.

Genetic testing has revealed possible MAFB mutations associated in MCTO.

Treatment with traditional anti-inflammatory drugs, conventional DMARDS, and biological therapies do not have any effect on the pain, progression of the diseases or the deformities caused.

Bisphosphonates are beneficial in improving acute pain, and bone mineral density in MCTO.

This clinical case teaches us to think outside the box and to revisit diagnosis for better patient outcome.

**Case report - Key learning points:**

JIA is a clinical diagnosis with a wide spectrum of differential diagnosis. Accurate diagnosis relies on clinical judgement. JIA mimics constitute a pitfall to even the most experienced clinician.

Atypical features such as persistently normal inflammatory markers, relative lack of pain or activity limitation despite swollen joints, should alert the physician to alternative diagnosis such as multicentric carpo-tarsal osteolysis.

Having a good knowledge about potential JIA mimics would help avoid unnecessary immunosuppression.

MCTO is a rare skeletal dysplasia which needs a high index of suspicion for diagnosis. Many questions still remain unanswered in this condition. Further research is needed to understand whether a high bone turnover is specific to MAFB gene mutation and if MCTO may represent a form of idiopathic juvenile osteoporosis in which short bones are more severely affected than long bones.

